# Experimental Diabetes Induces Structural, Inflammatory and Vascular Changes of Achilles Tendons

**DOI:** 10.1371/journal.pone.0074942

**Published:** 2013-10-09

**Authors:** Rodrigo R. de Oliveira, Conceição S. Martins, Yuri R. Rocha, Allysson B. R. Braga, Rômulo M. Mattos, Fábio Hecht, Gerly A. C. Brito, Luiz E. Nasciutti

**Affiliations:** 1 Department of Physical Therapy, Faculty of Medicine, Federal University of Ceará, Fortaleza, Brazil; 2 Department of Morphology, Faculty of Medicine, Federal University of Ceará, Fortaleza, Brazil; 3 Department of Morphology, Faculty of Medicine, Federal University of Ceará, Fortaleza, Brazil; 4 Department of Morphology, Faculty of Medicine, Federal University of Ceará, Fortaleza, Brazil; 5 Institute of Biomedical Sciences, Federal University of Rio de Janeiro, Rio de Janeiro, Brazil; 6 Department of Endocrinology, Federal University of Rio de Janeiro, Rio de Janeiro, Brazil; 7 Department of Morphology, Faculty of Medicine, Federal University of Ceará, Fortaleza, Brazil; 8 Institute of Biomedical Sciences, Federal University of Rio de Janeiro, Rio de Janeiro, Brazil; The Chinese University of Hong Kong, Hong Kong

## Abstract

This study aims to demonstrate how the state of chronic hyperglycemia from experimental Diabetes Mellitus can influence the homeostatic imbalance of tendons and, consequently, lead to the characteristics of tendinopathy. Twenty animals were randomly divided into two experimental groups: control group, consisting of healthy rats and diabetic group constituted by rats induced to Diabetes Mellitus I. After twenty-four days of the induction of Diabetes type I, the Achilles tendon were removed for morphological evaluation, cellularity, number and cross-sectional area of blood vessel, immunohistochemistry for Collagen type I, VEGF and NF-κB nuclear localization sequence (NLS) and nitrate and nitrite level. The Achilles tendon thickness (µm/100g) of diabetic animals was significantly increased and, similarly, an increase was observed in the density of fibrocytes and mast cells in the tendons of the diabetic group. The average number of blood vessels per field, in peritendinous tissue, was statistically higher in the diabetic group 3.39 (2.98) vessels/field when compared to the control group 0.89 (1.68) vessels/field *p* = 0.001 and in the intratendinous region, it was observed that blood vessels were extremely rare in the control group 0.035 (0.18) vessels/field and were often present in the tendons of the diabetic group 0.89 (0.99) vessels/field. The immunohistochemistry analysis identified higher density of type 1 collagen and increased expression of VEGF as well as increased immunostaining for NFκB p50 NLS in the nucleus in Achilles tendon of the diabetic group when compared to the control group. Higher levels of nitrite/nitrate were observed in the experimental group induced to diabetes. We conclude that experimental DM induces notable structural, inflammatory and vascular changes in the Achilles tendon which are compatible with the process of chronic tendinopathy.

## Introduction

Case reports and epidemiological studies suggest a relationship between Diabetes Mellitus (DM) and complications and ruptures of tendons from various regions of the human body [1], [2], [3], [4], [5], [6]. To evaluate this relationship in diabetic patients, studies have stopped at structural analysis of the tendon performed through diagnostic imaging methods, such as musculoskeletal ultrasound [1], [4], [5], [7], [8], computerized tomography [6] and magnetic resonance imaging [9]. A systematic literature review performed by our group pointed out that there is strong evidence of a relationship between DM and changes in tendon structure, such as, tendency for increased thickness, disorganization, alterations of the tendons and presence of calcification [10]. In addition to the occurrences of manifestations in the structure of tendons in patients with diabetes, recently, it has been reported that the mechanical properties of tendons in animals chemically induced to DM have undergone significant changes when compared to the control group [11], [12].

The classic characteristics of tendinopathy is intratendonous degeneration and disorganization in the composition of the extracellular matrix [13], [14], [15], [16] and vascular hyperplasia [17].

Recently, studies have identified vascular hyperplasia in painful tendons [18], [19], [20] and in degenerative change processes in tendons resulting from sports activity [21], [22], [23]. In addition, an increase of microvascular density, proliferation of endothelial cells and an increase in perivascular mast cells in tendinopathies have been confirmed [24], [25], [26] which demonstrates a strong link between vascular hyperplasia and tendon dysfunction caused by overuse or by microtrauma.

Despite several studies that associate chronic hyperglycemia with tendinopathy, it has not yet been established whether such vascular changes are also found in tendons of diabetics. Thus, the present study has as a hypothesis that Achilles tendons of experimental induced DM rats have characteristics of vascular, structural and inflammatory changes as in classical tendinopathy.

## Materials and Methods

### Animals

Rats with the following characteristics were used: albino, male *Wistar* rats (*Rattus Norvegicus*), weighing between 300 to 350 g, from Federal University of Ceará. The animals were kept in collective plastic cages (maximum of 5 animals/cage) in an environment with a temperature of 23±1°C, a light/dark cycle of (12 h) and with free access to maintenance diet (Labina® – Purina PetCare Company) and water *ad libitum*.

The procedures for handling and care of animals are in accordance with the international standards set by the *National Institute of Health Guide for Care and Use of Laboratory Animals* and were approved by the Commission of Ethics in Animal Experimentation – Federal University of Ceará/UFC, under protocol 51/2011.

### Experimental Groups and Induction of Diabetes

The animals were randomly divided into two experimental groups: control group – CG (n = 10) consisting of healthy rats; diabetic group – DG (n = 10) constituted by rats induced to DM.

The experimental diabetes, equivalent to Type I, was induced by intraperitoneal administration of streptozotocin (Sigma Chemical Co., USA) after fasting for 14 h. The streptozotocin (STZ) was diluted in 10 mM sodium citrate buffer at pH 4.5, in a single dose of 60 mg/kg animal weight, measured carefully with a precision digital scale. The control animals received, in the same way, equivalent doses of sodium citrate buffer solution, and after 30 min of treatment the animals were fed normally [27].

### Blood Sugar

Checking blood glucose occurred in the following stages of the experiment: 1– after the fast of 14 h leading up to the induction to diabetes; 2–7 days after induction, with the aim to meet the criteria for inclusion in the Diabetic Group since only animals that showed blood glucose above 200 mg/dL were included (Accu-Chek Activ Kit glucometer); 3– on the twenty-fourth day after induction to diabetes, aiming to evaluate the glycemic expression on the day of collection of the tendon. Reagent strips (Accu-Chek Activ) were used for determination of blood glucose from a drop of blood from the tip of the tail of the animals.

### Euthanasia and Collection of Samples of Achilles Tendon

On the twenty-fourth day after induction to DM, the animals of both groups were anesthetized with xylazine solution (Rompum® – Bayer) (10 mg/Kg) and ketamine hydrochloride (Ketalar®) (25 mg/kg), 0.10 mL for each 100 g body weight. After anesthesia an incision was performed in the back of the hind legs to collect the Achilles tendon from its origins and insertions. Subsequently, rats were euthanized by CO_2_ inhalation.

### Histological Evaluation of the Thickness Tendon, Vessels and Number of Cells

In the present study, the Achilles tendon was stained with H&E to verify the amount of blood vessels, measure the diameter of these vessels and for cell counting. For the count of Mast cells, preparations were stained with toluidine blue. By means of histological images stained with H&E obtained by the *LAS* software, counts were performed of the number of blood vessels of intratendinous and peritendinous regions and Mast cells in ten fields for slide in 6 slides per group (magnification 400x), from the *ImageJ* software. After open the histological field to be analyzed, the plugin *“Cell Counter”* was selected to quantify the blood vessels and cells, selected manually by the evaluator with the help of the mouse. The plugin tags and quantifies the different areas of interest are selected manually, posteriorly, the software does the count automatically. The measurement of tendon thickness is given by the average of three distances. The areas measured were previously marked in the distal, central and proximal regions of the tendons X400 viewing fields.

### Nitric Oxide Product

Nitric oxide formation was measured in serum samples by assaying nitrite/nitrate, one of the stable end-products of NO oxidation. Serum nitrite concentration was assayed spectrophotometrically using Griess reagents [1% sulfanilamide in 5% phosphoric acid and 0.1% N-1-naphthylethylenediamine dihydrochloride in bidistilled H_2_O (NED solution)] as described by Miranda et al. [28] A standard curve was run simultaneously with each set of samples.

### Immunohistochemistry

The tendon tissue was processed, kept in an oven at 60°C for 180 minutes, cleaned and bathed in xylene and gradients of alcohol. The tendon tissue was processed using the immunoperoxidase method. Immunohistochemistry was performed on an auto-assay machine – Autostainer Plus (Dako Diagnostics, Glostrup, Denmark) with Kit Ension Flex (Dako Diagnostics, Glostrup, Denmark). For antigenic recovery the tissue was exposed to 5% proteinase K (Sigma-Aldritch, Oakville, Canada) for 15 min, after which endogenous peroxidase was blocked with Envision Flex Bloking Serium for 10 min (Dako Diagnostics, Glostrup, Denmark). Subsequently, the samples were washed with buffer, then incubated for 60 min with the primary antibody VEGF – (Santa Cruz Biotechnology – mouse monoclonal antibody) diluted 1∶150 in standard diluent (Dako Diagnostics, Glostrup, Denmark), collagen type 1– (Santa Cruz Biotechnology – human monoclonal antibody) diluted 1∶50 in standard diluent (Dako Diagnostics, Glostrup, Denmark) and primary anti-NFκB p50 nuclear localization sequence (NLS; sc-114) (Santa Cruz Biotechnology – rabbit monoclonal antibody) diluted 1∶100 in standard diluent (Dako Diagnostics, Glostrup, Denmark). After washing, samples were incubated with Envision FLEX HRP (Dako Diagnostics, Glostrup, Denmark). For signalling a buffer substrate and Envision FLEX DAB Chromogen (Dako Diagnostics, Glostrup, Denmark) were used and counter-stained with Envision FLEX hematoxylin. Counts were performed of the number of cells immunostaing of the nucleus by NF-κB p50 NLS in ten fields for slide in 6 slides per group (magnification 400x), from the National Institutes of Health Image J software.

The slides were analyzed blindly from the groups using a 40X objective, with the tendon and peritendon scanned in their entirety and based on the number of fields with positive staining. For VEFG marking, each section was considered VEGF – positive or VEGF – negative. To be considered VEGF – positive, specific marking had to be present in three or more captured fields of vision, making sure that the sections with only one or two positive areas were not considered positive. For quantification of DAB-stained of Type 1 collagen and VEGF, five 400 magnification pictures from each group were assessed with Computer-assisted Methods of Quantifying Brightfield Microscopy Images of the National Institutes of Health Image J software.

### Statistical Analysis

To describe the characteristics of the sample, descriptive measures were used, such as: measure of central tendency (mean) and dispersal (standard errors). For comparison of the averages of numerical variables between the various treatments employed, the *Student t-test*was used for analysis of independent samples for comparison between the control group and diabetic group. To assess the presence or absence of VEGF, the Fisher exact test was used. The data were analyzed with *SPSS* software (*Statistical Package for Social Sciences*). The significance level was set at 5%.

## Results

**Figure 1:**
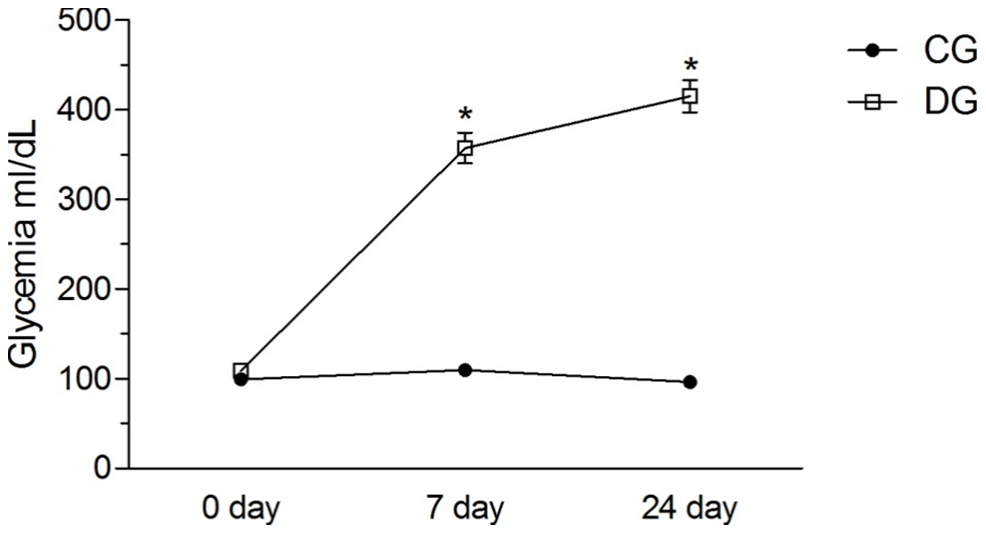
Glycemic Expression ml/dL at three evaluations. CG – Control group and DG – Diabetic group. *- *p* = 0.001. *Student t* test to determine statistical differences of CG and DG.

The blood sugar levels of the control animals remained stable during all the analyses, however, the animals induced to DM group presented a significant increase in the measurements after the induction of DM in the seventh and the twenty-fourth day (*p* = 0.001) – Figure 1. Presentation of the animal weight and thickness of the tendons are presented in Table 1.

**Table 1:** Presentation of the animal weight and thickness of the tendons.

Characterization of the sample (24 days old)		
Variable	control group	diabetic group
Weight (g)	361.2 (3.3)	221.7 (2.5)*
Thickness Tendon (µm)	433.0 (5.1)	341.1 (6.5)*
Thickness Tendon (µm/100 g)	119.8 (1.3)	153.8 (1.0)*

Statistical analysis and comparison of means for independent samples. Student t test values expressed as mean (X), standard error of the mean (SEM). *p<0.05.

### Thickness and Cellularity

**Figure 2:**
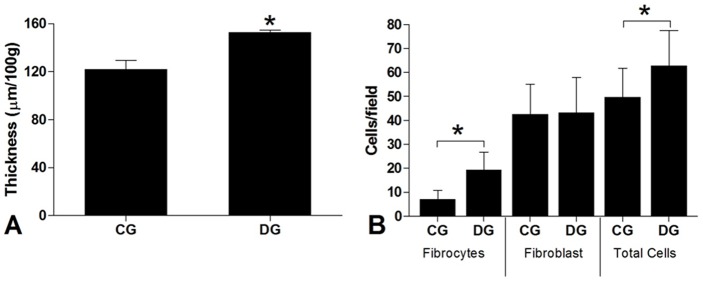
Thickness of the Achilles tendons in the Control Group – CG and the Diabetic Group – DG with results are exhibited in terms of thickness value/100 g of the animal's weight. **A**. Quantification of cell density – Fibrocytes, fibroblast and Total Cells in the Control Group – CG and the Diabetic Group – **B**. H.E sections (10 most proximal X400 viewing fields).The values are expressed as means and standard errors. * – p<0.05 Student-t Test for independent samples showing statistical differences between the groups studied.

The Achilles tendon thickness of diabetic animals showed, in an analysis by thickness, an important increase when compared to the control group – Figure 2-A. Similarly, an increase was observed in the density of fibrocytes and total cellularity in the tendons of the diabetic group – Figure 2-B.

### Blood Vessels

**Figure 3:**
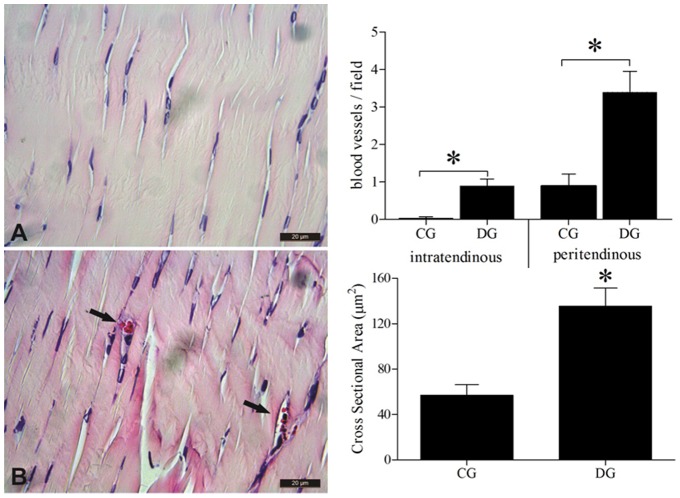
Density and Cross Sectional Area of blood vessels in the Achilles tendon in the Control Group – CG and the Diabetic Group – DG in the intratendinous and peritendinous regions. The values are expressed as means and Standard Deviation of the Mean (SEM). * –p<0.05 Student-t Test for independent samples between the groups studied. Normal tendon –**A;** Tendon of the diabetic group with arrows pointing to blood vessels –**B**. 400X – HE.

The blood vessels were present in peritendinous tissue of both groups, however, the average number of blood vessels per field was statistically higher in the diabetic group 3.39 (2.98) vessels/field when compared to the control group 0.89 (1.68) vessels/field *p* = 0.001. In assessing the average numbers of blood vessels in the intratendinous region, it was observed that blood vessels were extremely rare in the control group 0.035 (0.18) vessels/field and were often present in the tendons of the diabetic group 0.89 (0.99) vessels/field, and presented statistical difference *p* = 0.001– Figure 3.

The blood vessels in the Achilles tendons of the DG showed increasing cross-sectional area, with vessels reaching an average of 135.58 (15.77) µm^2^ while the healthy group reached 56.99 (9.33) µm^2^, and presented statistical difference *p* = 0.001– Figure 3.

### Density of Mast Cell

**Figure 4:**
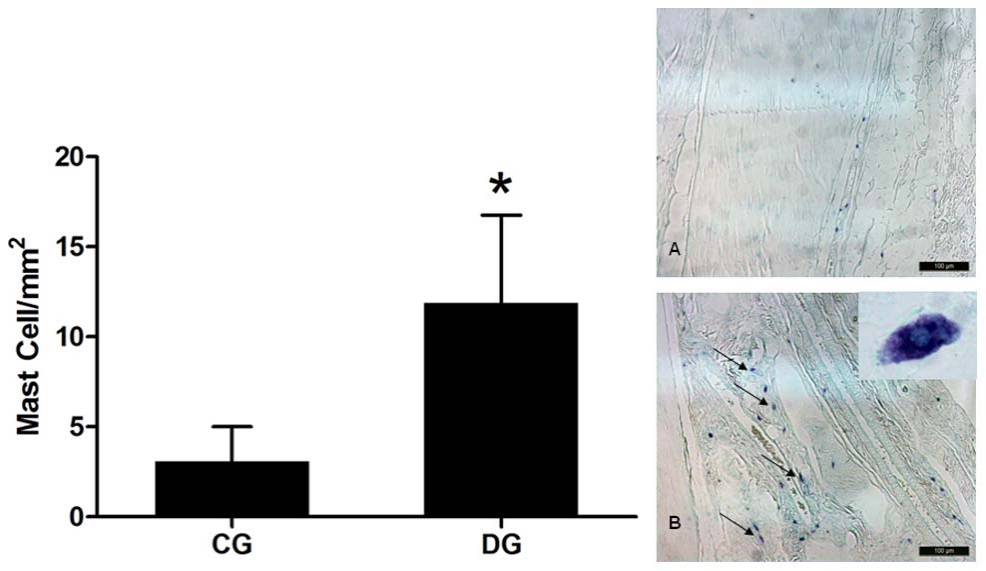
Density of mast cells in the Achilles tendon in the Control Group – CG and the Diabetic Group – DG. **Normal tendon. A;** Tendon of the diabetic group with arrows pointing to mast cells –**B**. * –p<0.05 Student-t Test for independent samples between the groups studied –400X – Toluidine blue.

The number of Mast cells prevailed in tendons of diabetic animals which had a density of 11.90 (4.86) cells/mm2, while the control group showed a density almost 4 times less dense, being 3.10 (1.91) cells/mm2 – Figure 4. Mast cells that were present in the DG were distributed mostly close to blood vessels.

### Nitric Oxide Product

**Figure 5:**
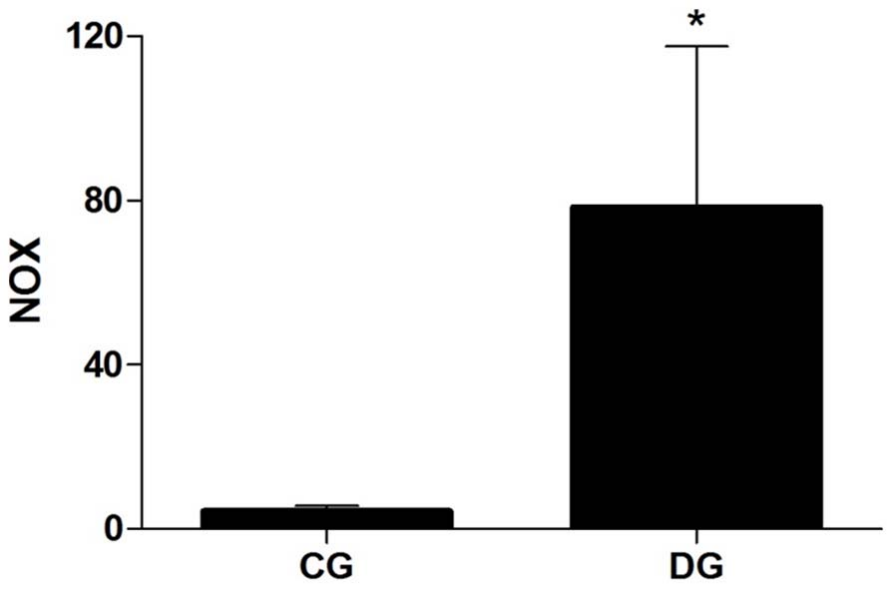
Level of nitric oxide product – nitrite/nitrate (NOx) in the Achilles tendon in the Control Group – CG and the Diabetic Group – DG. * – p<0.05 Student-t Test for independent samples between the groups studied.

To evaluate the production estimates of nitric oxide the levels of nitrite/nitrate (NOx) were quantified. Higher levels of nitrite/nitrate (NOx) were observed in the experimental group induced to diabetes when compared with the control group (p<0.05). – Figure 5.

### Type 1 Collagen

**Figure 6:**
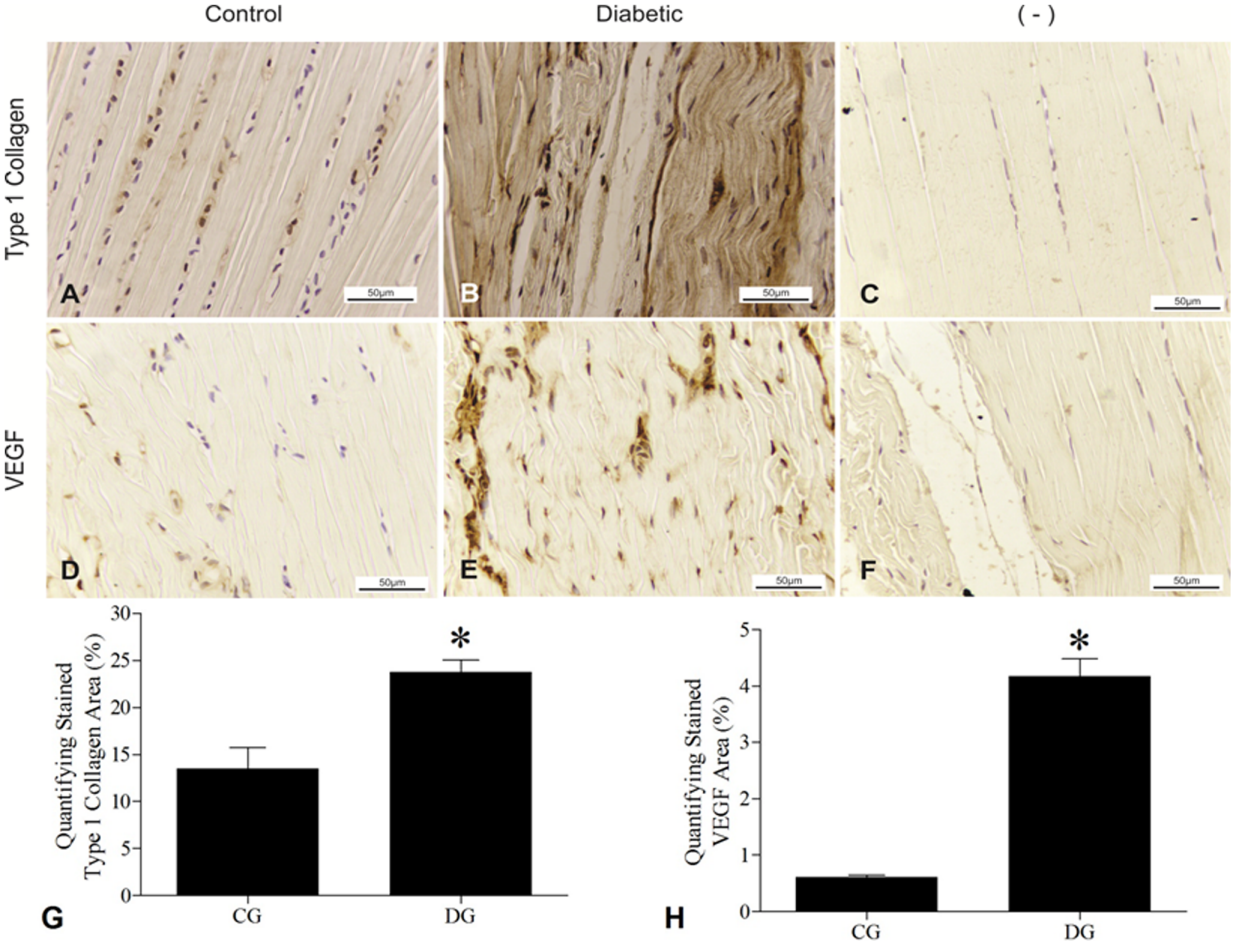
Immunomarking the density of Type I Collagen and VEGF. Density of type I Collagen in the Achilles tendon in the Control Group. **A;** Increased of the density of type I Collagen in the Achilles tendon in the Diabetic Group and disorganization in the Extracellular Matrix –**B**; The absence of VEGF expression in the control group –**D**; Expression of VEGF in the diabetic group –**E**; Negative Controls –**C** and **F**. 400X. Relative stained area was quantified using National Institutes of Health ImageJ software. The bar graph summarizes average values of each group for type 1 collagen –**G** and VEGF –**H**. The values are expressed as means and Standard Deviation of the Mean (SEM). * –p<0.05 Student-T Test for independent samples showing statistical differences between the groups studied.

The immunohistochemistry analysis identified areas of higher density of type 1 collagen, associated with the disorganization of the fibers in the diabetic group when compared to the control – Figure 6-A, B and G–*p* = 0.003.

### Expression of VEGF

Expression of VEGF was found in all Achilles tendons of the diabetic group, while there was no marking in any tendon of the healthy group –*p* = 0.001 – Figure 6- D, E and H

### Expression of NF-κB

**Figure 7:**
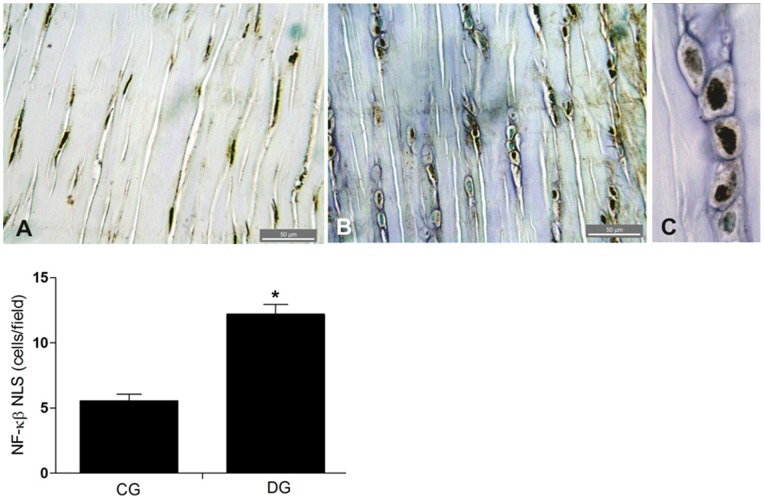
A greater degree of Immunomarking of NF-κB nuclear expression was observed in the diabetic group. **B;** when compared to the control group – **A**. Highlights of immunomarking of NF-κB nuclear expression in the diabetic group – **C**. *-*p* = 0,001 Student-t Test for independent samples between the groups studied.

Increased immunostaining for the NFκB-p50 NLS (which specifically binds to the dissociated fraction p50 of NFκB in the nucleus or in the cytoplasm) was detected in Achilles tendon of the diabetic group when compared to the control group – Figure 7. This was confirmed in the cell count of the nuclear expression of NF-κB by field, where the group with experimental diabetes obtained an average represented by 12.21 (0.74) marked cells by field, while the healthy group expressed 5.57 (0.49) – (p = 0.001) – Figure 7.

## Discussion

This study aims to demonstrate how the state of chronic hyperglycemia from experimental DM can influence the homeostatic imbalance of tendons and, consequently, lead to the characteristics of tendinopathy. To this end, the Achilles tendon was chosen, due to its important role and its superficial location. In addition, some studies have suggested biomechanical dysfunction and histological changes of the Achilles tendon of animals induced to experimental diabetes [11], [29].

Here, it was demonstrated that the diabetic group showed significant increase in average thickness of the Achilles tendon when compared to the control group. Several other studies had already shown that the state of chronic hyperglycemia triggers a process of increased thickness of the tendon [1], [4], [5], [6], [10], [30].

Among the factors that may contribute to thickening of the tendon is the increased production of collagen and other extracellular matrix components, which in turn are deposited in a disorganized manner as demonstrated in this study. This increase can be related to the increased cellularity found here in diabetic tendons. These cells are involved in a cyclical process with excessive deposition of collagen fibers in the tissue, causing tissue fibrosis, which compromises the extracellular matrix and leads to cell death [31].

The Achilles tendons in the diabetic group showed vascular responses uncommon to healthy tendons, such as increased number of blood vessels, vascular migration to the central tendon and increase in cross-sectional area of vessels. The number of blood vessels in the peritendinous region of the Achilles tendons in the DG is higher than that found in the control group, and in the intratendinous region the presence of vessels proved very frequent in diabetic animals and very rare in tendons of healthy animals. These findings are taken as characteristic of chronic tendinopathies and have already been demonstrated recently by a study with Doppler ultrasonography in tendons of patients with type 2 DM [32] and by various other studies with symptomatic tendons and those that have suffered injury by overuse [18], [20], [21], [22].

Vascular hyperplasia, being one of the classic features of chronic changes of the tendons, is one of the causes of pain due to parallel migration of vascular innervation to the high tension, mechanical region of tendon [18], [33], [34], and can also contribute to the thickening and disorganization of the tendon. repair process inducing inflammation will lead to increased metabolic demand and, consequently, may trigger increased vascular density [35].

It was also detected an increase in number of mast cells in the Achilles tendons of diabetic group. This finding was also reported by another original study that evaluated the density of mast cells in patellar tendons of individuals who had symptomatic chronic tendinopathy for more than three months when compared with tendons of healthy individuals [25]. Furthermore, the occurrence of increased population of mast cells was reported in deep flexor tendons of the legs of rabbits that were in the process of healing and fibrosis [36], as the increase of mast cells is related to the event of soft tissue remodeling after chronic injury, with the function of releasing profibrotic factors and tryptase that shape this process [25], [37].

Faced with a chronic stress in tendons the mast cells release angiogenic growth factors which stimulate the proliferation of new blood vessels [25]. However, in this study, the relationship of the increment of mast cells with hypervascularization was not evaluated, but, the proximity of these cells to blood vessels of animals with DM was observed.

We describe the increase in vascular density, vascular hyperplasia and the increment of mast cells in tendons of the diabetic group. Vascular endothelial growth factor (VEGF) may have a role in these processes, as the diabetic group showed high expression of VEGF in all Achilles tendons, which did not occur in tendons of the control group. VEGF is a growth factor not expressed in mature and healthy tendons [38]. However, it is expressed in tendons in chronic degeneration [39].

The expression of VEGF is an element of the recognition response to soft tissue injury, being induced by cytokines, increased mechanical load and hypoxia [40]. However, it is believed that the increase of VEGF expression in diabetic animals is related to the advanced glycated end products (AGEs) present in states of chronic hyperglycemia. The interaction of AGE with its receptor (RAGE) in endothelial cells activates NF-κB transcription, leading to the subsequent increased expression of their target genes, such as VEGF [41].

The increased activation of the transcription factor NF-κB is present in several complications of DM, such as retinopathy [42], cardiomyopathy [43] and muscle changes [44], [45]. The results of this study also demonstrated increased activation of NF-κB detected by immunostaing of the nucleus by NF-κB p50 NLS (nuclear localization sequence) in the tendinocytes of the group with DM.

**Figure 8:**
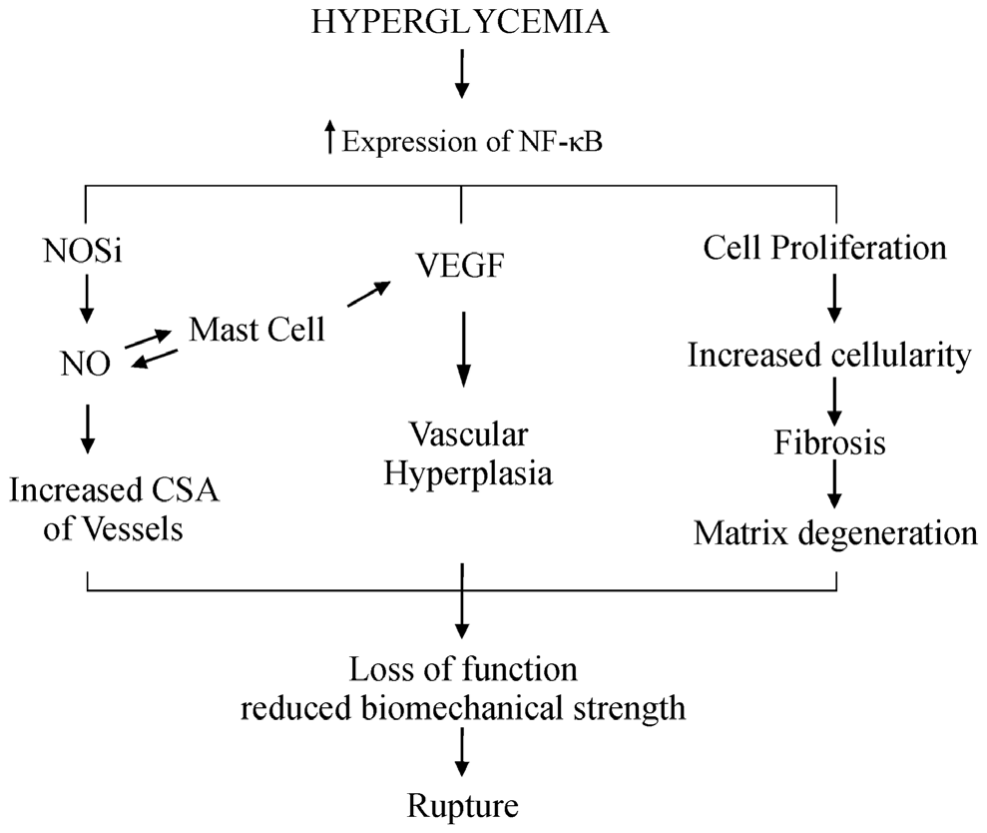
Signaling pathways in diabetic tendinopathy. Initially, the state of chronic hyperglycemia results in the activation of NF-κB, which leads to the increased expression of target genes such as VEGF and NOSi. These, in turn, result in increased NO and vascularization. This increased vascularization associated with cell proliferation and possible migration causes hypercellularity and the rise of disorganized deposition of type 1 collagen. Mast cells increased as a result of an inflammatory process of the tendon denoted by the increase of nitrite and nitrate indicative of increased NO also contribute to the increase of VEGF and therefore to increased vascularity.

In an *in vitro* experiment, was demonstrated that the expression of NF-κB increases at faster rates until 8 weeks after tendon injury [46]. As already described, this increase in NF-κB is probably involved in regulating the expression of target genes during healing of the tendon. Based on the results of this and previous studies, we propose a response pathway to diabetic tendinopathy. Initially, the state of chronic hyperglycemia results in the activation of NF-κB, which leads to the increased expression of target genes such as VEGF and NOSi. These, in turn, result in increased NO and vascularization. This increased vascularization associated with cell proliferation and possible migration causes hypercellularity and disorganized deposition of type 1 collagen. The inflammatory process as detected by nitrite and nitrate indicative of increased NO release, may be related to greater counting of mast cells, which also contribute to the increase of VEGF and therefore to increased vascularity. These events, if maintained, lead to the loss of function and reduction of biomechanical properties of diabetic tendons (Figure 8).

Although this study evaluates the relationship of DM with vascular changes of the Achilles tendon across the board, it does not establish causality of the phenomena of increased mast cells, vascular hyperplasia and increased expression of VEGF and NF-κB in Achilles tendons of this population. However, based on the results of the studies previously cited and, mainly, by the experiments performed in this study, one can establish that the diabetic group presents a picture of chronic diabetic tendinopathy of the Achilles tendon. This condition may lead individuals to additional morbidities such as the inability to perform physical activities, increased plantar pressures, eventual attenuation of the anterior tibial tendon and rupture of the Achilles tendon itself [11].

## Conclusion

Briefly, we conclude that the group induced to DM presented alterations to the Achilles tendon with structural and vascular changes that are compatible with the process of chronic tendinopathy, since, in the presence of hyperglycemia important changes occurred, such as an increment of mast cells, significant increase in the number of blood vessels in the peri- and intratendinous regions, as well as increased cross-sectional area of the vessels and increased expression of type 1 collagen, VEGF and NF-κB when compared to tendons of healthy animals. Further studies are needed to assess the cause-and-effect relationship of tendinous manifestations with the state of hyperglycemia.
